# Value of handcrafted and deep radiomic features towards training robust machine learning classifiers for prediction of prostate cancer disease aggressiveness

**DOI:** 10.1038/s41598-023-33339-0

**Published:** 2023-04-17

**Authors:** Ana Rodrigues, Nuno Rodrigues, João Santinha, Maria V. Lisitskaya, Aycan Uysal, Celso Matos, Inês Domingues, Nickolas Papanikolaou

**Affiliations:** 1 Champalimaud Research, Champalimaud Foundation, Lisbon, Portugal; 2 Faculty of Medicine, University of Porto, Porto, Portugal; 3 LASIGE, Faculty of Sciences, University of Lisbon, Lisbon, Portugal; 4 Instituto Superior Técnico, University of Lisbon, Lisbon, Portugal; 5 Cand. of Sci. (Med.), Radiologist at Radiology Department with CT and MRI, Medical Research and Educational Center, Lomonosov Moscow State University, Moscow, Russia; 6 Gulhane Medical School, University of Health Sciences, Ankara, Turkey; 7 Instituto Politécnico de Coimbra, Instituto Superior de Engenharia, Rua Pedro Nunes-Quinta da Nora, 3030-199 Coimbra, Portugal; 8 Centro de Investigação do Instituto Português de Oncologia do Porto (CI-IPOP): Grupo de Física Médica, Radiobiologia e Protecção Radiológica, Porto, Portugal

**Keywords:** Computational biology and bioinformatics, Oncology

## Abstract

There is a growing piece of evidence that artificial intelligence may be helpful in the entire prostate cancer disease continuum. However, building machine learning algorithms robust to inter- and intra-radiologist segmentation variability is still a challenge. With this goal in mind, several model training approaches were compared: removing unstable features according to the intraclass correlation coefficient (ICC); training independently with features extracted from each radiologist’s mask; training with the feature average between both radiologists; extracting radiomic features from the intersection or union of masks; and creating a heterogeneous dataset by randomly selecting one of the radiologists’ masks for each patient. The classifier trained with this last resampled dataset presented with the lowest generalization error, suggesting that training with heterogeneous data leads to the development of the most robust classifiers. On the contrary, removing features with low ICC resulted in the highest generalization error. The selected radiomics dataset, with the randomly chosen radiologists, was concatenated with deep features extracted from neural networks trained to segment the whole prostate. This new hybrid dataset was then used to train a classifier. The results revealed that, even though the hybrid classifier was less overfitted than the one trained with deep features, it still was unable to outperform the radiomics model.

## Introduction

In 2020, prostate cancer was the second most frequent cancer in men worldwide and ranked 5th in terms of mortality, being the leading cause of death in 48 out of 185 countries as analysed by Sung et al.^1^. Prostate cancer diagnosis procedure relies on unspecific measures such as PSA (prostate-specific antigen) levels and DRE (digital rectal examination), followed by biopsy^2^, where disease aggressiveness assessment is based on the Gleason Score. This biomarker is used to define the clinical significance of lesions, according to which treatment decisions are made. Thus, an accurate determination of clinical significance is essential for ascertaining the most appropriate treatment options and ensuring the best clinical outcome.

Recent developments in Artificial Intelligence anticipate much needed improvements in the detection, diagnosis, screening, and staging of prostate cancer^3^. One area of particular interest is radiomics, which allows for quantitative analysis of medical images, contrary to the qualitative analysis performed by experts in the field thus far. Radiomics is defined as the transformation of medical images into high-dimensional mineable data in the form of extracted quantitative features^4^. Studies using radiomics have shown potential since the features’ quantitative nature eliminates some of the inherent subjectiveness of medical image interpretation. Moreover, radiomics is able to turn radiological images into tabular data, which machine learning algorithms can later analyse^5^. The latter are designed to detect patterns in the data and are able to find useful diagnostic and prognostic biomarkers that would not be seen by the naked eye of an expert radiologist. The use of radiomic features extracted from bpMRI (bi-parametric Magnetic Resonance Imaging) exams to predict prostate cancer disease aggressiveness can be found in the literature^6–14^.

Radiomics, however, has several shortcomings. A major limitation is the tight link between the computed radiomic features and the volume of interest (VOI) from where they have been extracted. Tissue or lesion segmentation is performed either manually, when a radiologist outlines the boundary of the VOI in the image, automatically, for example by a deep learning algorithm trained to segment a certain VOI, or semi-automatically, where the mask is drawn in an automated fashion and later verified and corrected by a radiologist. When manually traced, the segmentation masks suffer from inter- and intra-reader variability^15^. These slight real-world differences in human-defined segmentation margins may in principle affect the the distribution of the calculated radiomic features, which subsequently affect the algorithms trained with them^16^. Building machine learning algorithms that are robust to this real-world heterogeneity is essential to a future safe application of AI methods in the clinical setting.

Parallel to the growing interest in radiomic features, deep Neural Networks have emerged as a promising technique for prostate cancer detection and segmentation of anatomic zones or tumorous lesions^17^. However, it has been shown that deep learning models tend to overfit when attempting to solve prostate cancer classification problems, not generalizing to out of distribution data^18^. Despite the large number of studies published, very few compare the performance of handcrafted radiomic features and deep features on the same data and objective^18,19^.

Regarding the classification of prostate cancer disease aggressiveness, we attempted to answer two research questions. Firstly, we compared different approaches and obtained insights into how to produce classifiers that are robust to differences in segmentation margins. And secondly, we have not only compared the performance of radiomic features and deep features for the classification of prostate cancer aggressiveness but also assessed the performance of models trained with hybrid datasets incorporating both handcrafted radiomic and deep features.

## Research questions

In this section, we will describe the research questions addressed in this study.

### Research question I (RQ I)

*Which is the best approach to train robust classifiers to minor differences in segmentation margins derived from two radiologists?*

**Figure 1:**
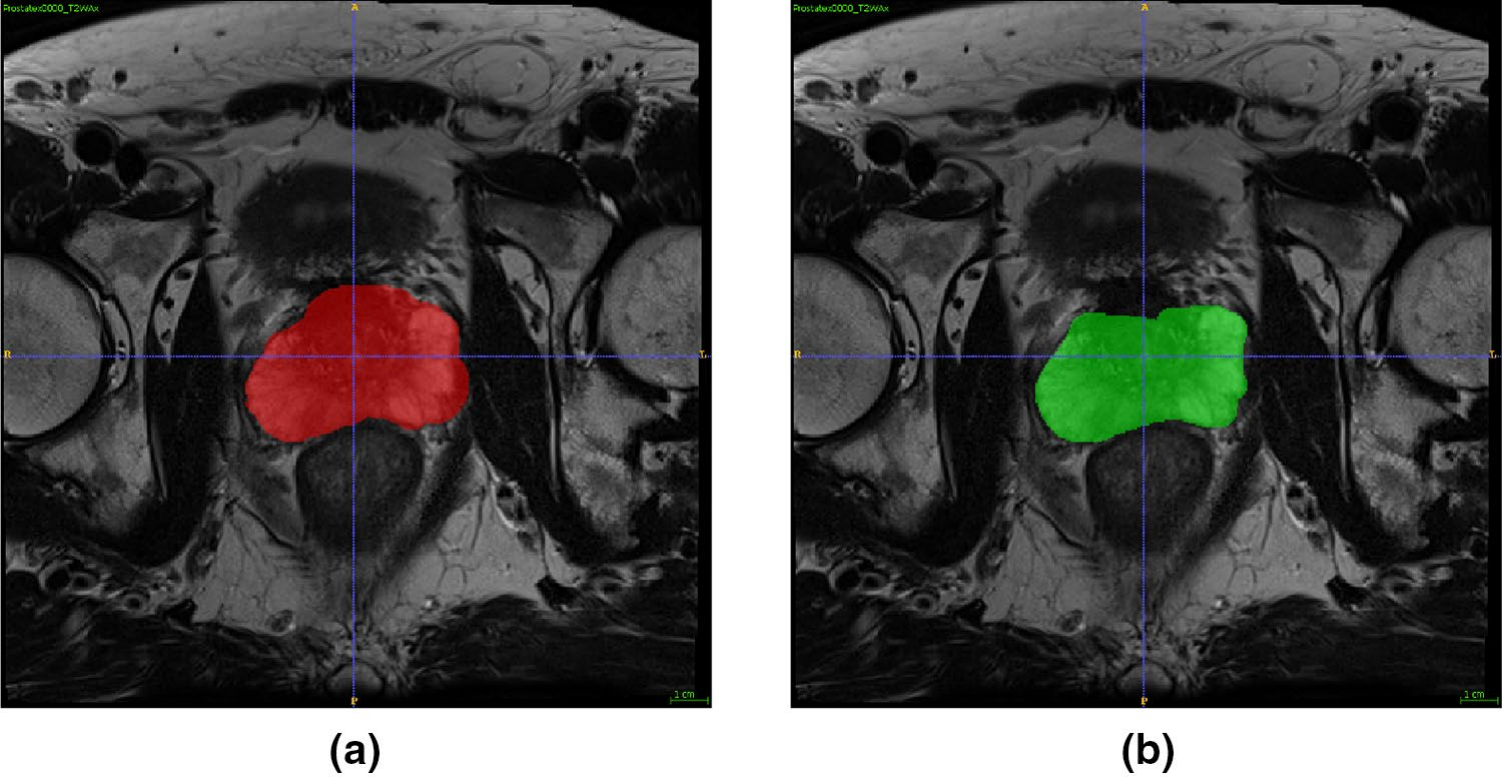
An example of the evident segmentation variability in the masks drawn by radiologist 1, (**a**), and radiologist 2, (**b**), on patient Prostatex0000.

An example of the common differences in segmentation margins can be found in Fig. 1. To answer RQ I, we compared different approaches at training:

$$\mathbf{1}{\mathbf{st}}$$ **approach**
(**stableRad1**) Perform a feature stability analysis and train only with stable features.
$$\mathbf{2}{\mathbf{nd}}$$ **approach**
(**Rad1**) Train with features extracted from masks drawn by radiologist 1 (rad1).
$$\mathbf{3}{\mathbf{rd}}$$ **approach**
(**Rad2**) Train with features extracted from masks drawn by radiologist 2 (rad2).
$$\mathbf{4}{\mathbf{th}}$$ **approach**
(**avgRad**) Train with the feature average between both radiologists.
$$\mathbf{5}{\mathbf{th}}$$ **approach**
(**intersectionRad**) Train with features extracted from the intersection of the two masks.
$$\mathbf{6}{\mathbf{th}}$$ **approach**
(**unionRad**) Train with features extracted from the union of the two masks.
$$\mathbf{7}{\mathbf{th}}$$ **approach**
(**resampledRad**) Train with a randomly resampled dataset where for some patients the extraction was performed from the mask drawn by rad1 and for others the extraction was performed from the mask drawn by rad2.

The dataset that resulted in the most robust classifier was selected for further analysis (research question II).

### Research question II

*Can deep features significantly improve the performance of machine learning classifiers trained with handcrafted radiomic features?*

To answer research question II, three approaches were compared for model training. These included a handcrafted radiomics dataset, a dataset with “deep” features, and a hybrid dataset with both.

## Methods

### Data description

Our dataset consisted of T2W, DW, and ADC data from the SPIE-AAPM-NCI PROSTATEx challenge^20–22^. The MRI exams were acquired at the Prostate MR Reference Center—Radboud University Medical Centre in the Netherlands. Due to the public nature of the data, ethics committee approval and patient consent were waived for this study. The dataset is composed of 181 patients. Due to complications with some segmentation files, we did not utilize the full extent of the PROSTATEx dataset. The full list of excluded patients can be found in Supplementary Material (SM 1). The approximate location of the centroid of each lesion was provided in DICOM coordinates. Cancer was considered significant when the biopsy Gleason score was 7 or higher. The lesions were labelled with “TRUE” and “FALSE” for the presence of clinically significant cancer, with a distribution of 67 clinically significant lesions (TRUE) and 214 clinically non-significant lesions (FALSE). A gland was considered to have clinically significant cancer if at least one of its lesions is clinically significant for prostate cancer. This resulted in a label distribution of 122 clinically insignificant glands and 59 clinically significant glands, giving a total of 181 patients.

### Methods specific to RQ1

#### Segmentation

Manual segmentations of the whole prostate gland were performed independently by two radiologists (M.L., 10 years of experience, and A.U., radiology resident) on T2W and DWI high b-value images separately.

#### Radiomic features extraction

Bias field correction was performed on T2W images using the N4 Bias Field Correction algorithm^23^ and the Python package Simple ITK (version 2.0.0)^24^. First, each image’s x-, y- and z-spacing was checked for discrepancies. Since x- and y-spacings differed from z-spacing, feature extraction was later performed in 2D. Additionally, T2W images’ x- and y-spacings differed within and between patients, so these were resampled to the highest value of 0.5. Non-quantitative images’ (T2W and DWI) intensities were normalized. The bin width was selected to produce discritized images with between 30 and 130 bins. This resulted in a bin width of 20 for T2W images, 5 for DWI, and 70 for ADC maps.

Radiomic features were extracted from the whole gland segmentation using the Pyradiomics package (version 3.0)^25^ in Python (version 3.7.9)^26^. All the pre-processing steps mentioned before were performed as parameters of the extractor function, except for the bias field correction, which was performed prior to the extraction. All image filters and feature classes were enabled, resulting in a total of 3111 features extracted, 1037 from each MRI modality (T2W, high b-value DWI and ADC). In the feature extraction of the ADC map, the mask drawn on the DWI was used. The mathematical expressions and semantic meanings of the features extracted can be found at https://pyradiomics.readthedocs.io/en/latest/.

#### Spatial stability of radiomic features

For approach 1, spatial stability was assessed by comparing the features extracted from the VOIs created by each radiologist. This analysis was conducted with a two-way, single rater, absolute agreement Intraclass correlation coefficient (ICC) formulation (ICC 2.1)^27^. Features with ICC 95% confidence interval lower limit over 0.75 were considered to be robust to segmentation and were kept for further analysis.

### Methods specific to RQ2

On top of the radiomic features utilized in RQ1, to answer RQ2, we also extracted deep features.

#### Deep features extraction

Deep features were extracted from segmentation models trained to segment the whole gland. To train the network, the volumes were cropped on both the X and Y axis, and zero padded on the Z axis. A comprehensive description of the training as well as network performance can be found in previous work^28^. The ground truth masks used for the gland were obtained as described previously, while both the peripheral and transitional zone masks were the publicly available ones from the SPIE-AAPM-NCI PROSTATEx challenge.

The models used to perform the segmentations were all Encoder-Decoder Unet variations, namely: Unet^29^; Unet++^30^; Attention Unet (AUnet)^31^; Dense Attention Unet (DUnet); Dense-2 Unet (D2Unet)^32^; Dense-2 Attention Unet (D2AUnet); Recurrent Residual Unet (R2Unet)^33^; Recurrent Residual Attention Unet (R2AUnet).

**Figure 2:**
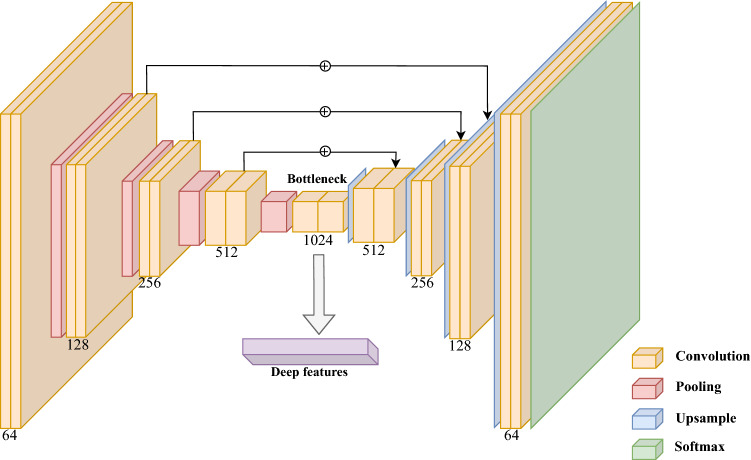
Representation of the deep feature extraction procedure from the segmentation models. These features are extracted from the bottleneck of the encoder-decoder model following three different strategies.

Taking these segmentation models, we removed the decoder part of the network and devised three different strategies to extract the deep features from the bottleneck (Fig.  2). The first approach consisted of averaging all the feature maps into a single one before flattening it, producing 200 features. For the second and third approaches, we removed all spatial dependencies of the feature maps by performing pooling operations, one version with Max-pooling and another Average-pooling, per feature map. This reducing each feature map to a single value, which were then concatenated, producing sets of 1024 features. The 8 different neural networks described were combined with the 3 different strategies for calculating the features, resulting in 24 deep datasets. In addition, the best radiomics dataset, selected in RQ I, was combined with the different whole gland deep features datasets to produce another 24 hybrid datasets.

### Train/test split

The train/test split was performed at a patient level with the Python scikit-learn package (version 0.23.2)^34–36^. The hold-out test set consisted of 33% randomly selected patients and the split was stratified so that both train and test sets have the same proportion of True labels. The patient list of the original split was used to split the remaining datasets, ensuring that the patients belonging to the hold-out test set did not differ between datasets and avoiding data leakage. The hold-out test set comprised of 58 patients.

### Dataset pre-processing

All the steps described in this section were performed on the train set.

Features with zero or near-zero variance were identified and excluded with the nearZeroVar method of the R caret package (version 6.086)^37^.

Feature correlation was then assessed. Feature pairs were considered correlated if their Spearman correlation was higher than 0.75. Out of the two, the feature with the highest average correlation across all features was eliminated.

Feature values were scaled to have 0 mean and standard deviation equal to 1.

To cope with the imbalanced nature of the data, the SMOTE (synthetic minority oversampling technique)^38^ algorithm was applied.

Finally, due to the high-dimensional feature space, we also explored a computationally “light” tree-based feature selection algorithm, where features with the highest tree importance were selected. The number of features selected was a hyperparameter optimized during model training.

### Model development

The scikit-learn implementation of two machine learning algorithms was used: Random Forest Classifier^39^ and Logistic Regression. These were chosen since they are on opposite ends of the bias-variance spectrum. Hyperparameter tuning was performed for each algorithm with an exhaustive grid search and each parameter combination was evaluated through cross-validation. The classifiers’ probabilities were calibrated (method = isotonic) using the Python scikit-learn package (version 0.23.2)^34–36^.

### Model evaluation

**Figure 3:**
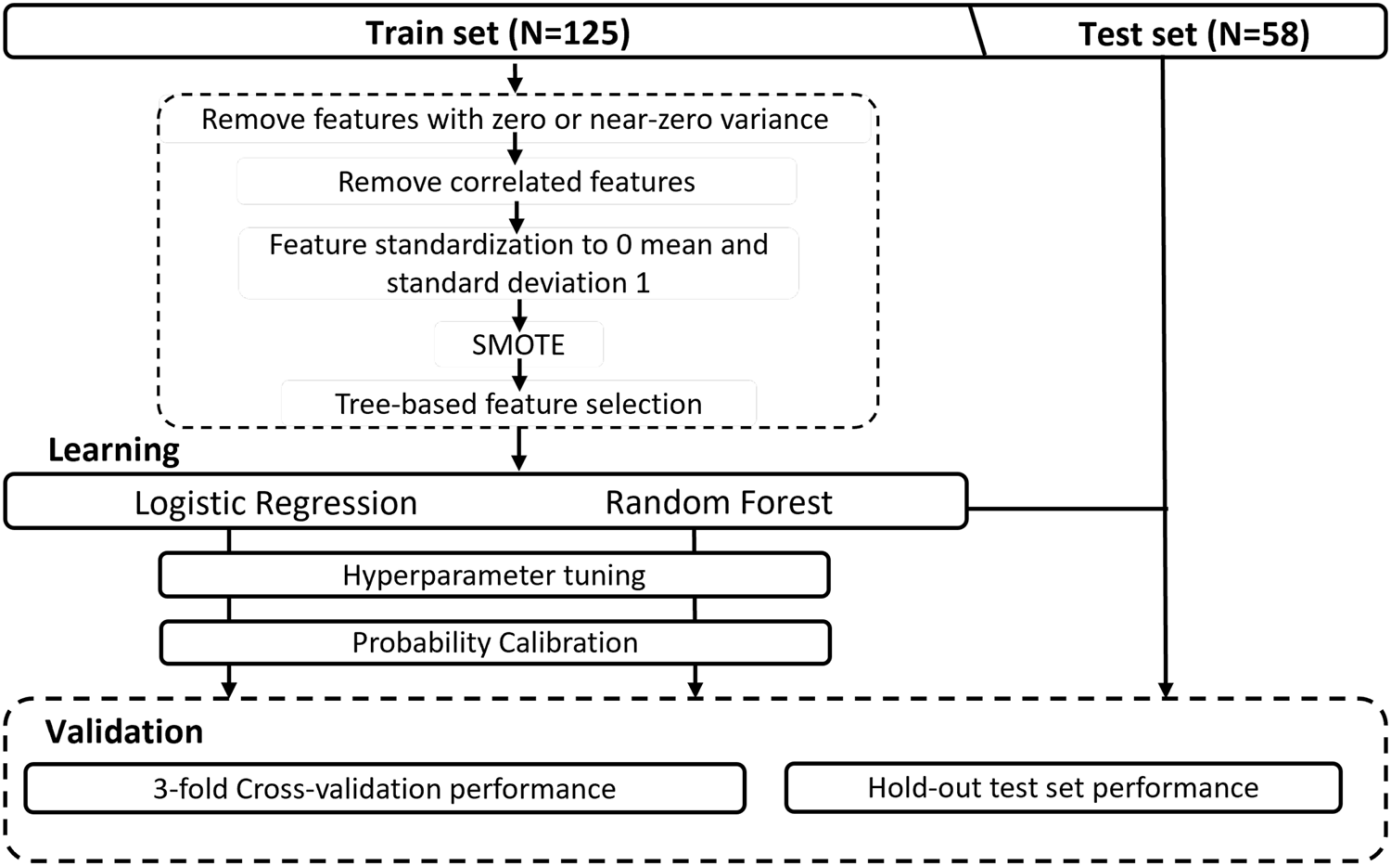
Overall model development pipeline followed in this study.

The probabilistic decision threshold for binary classification was chosen to achieve at least 90% sensitivity on the training set. For that reason, the area under the receiver operating characteristics curve (AUC) was used for hyperparameter tuning and pipeline selection since it is a metric invariable to the decision threshold. The pipelines were validated internally through 3-fold cross-validation, and the one with the highest cross-validation AUC was selected for each approach. For RQ I, the selected classifiers were applied to three hold-out test sets: each independent radiologist’s test set (approaches 2 and 3) and the resampled radiologists’ test set (approach 7). These hold-out test sets were chosen for being the ones that resemble real-world scenarios the most. For RQ II, the classifiers were applied to the hold-out test set respective to the same dataset as used for training. The overall pipeline followed in this study can be found in Fig. 3.

### Statistical comparison of classifiers

The classifiers selected for each approach were compared with McNemar’s test implemented by the mlextend python package (version 0.21.0)^40^. P-values were corrected for multiple comparisons with the FDR method and statistical significance was considered at $\alpha =0.05$ for all tests.

### SHAP importance of predictive variables

A SHapley Additive exPlanations (SHAP) analysis^41^ was used to identify the most relevant variables for the prediction. This was completed using the Python package shap (version 0.38.1).

## Results

### Research question I

#### Hold-out test set performance

For five of the approaches, the selected pipeline included Random Forest, while for the remaining approaches, 1 and 5, it performed better whilst using Logistic Regression. The training hyperparameters, the decision threshold, and the number of features can be found in the supplementary material (SM 2).

Applying the selected classifiers to rad1, rad2, and resampledRad hold-out test sets, Table 1a–c), respectively, we can see that the resampledRad dataset consistently produces the highest performing classifiers, following approach 7. On the contrary, the lowest-performing models were obtained by approach 1, which consisted of training classifiers only with features robust to segmentation differences.

**Table 1:** Classification performance on three different hold-out test sets.

(a)					
Training data	rad1 Hold-out test-set performance				
	F2	CohensKappa	AUC	Sensitivity	Specificity
stableRad1	0.6696	0.2042	0.7625	0.7895	0.4615
Rad1	0.7353	0.4636	0.7827	0.7895	0.7179
Rad2	0.7143	0.5110	**0.8219**	0.7368	**0.7949**
avgRad	0.6881	0.2758	0.7584	0.7895	0.5385
unionRad	0.7522	0.3073	0.7814	**0.8947**	0.4872
intersectionRad	0.6364	0.1863	0.6802	0.7368	0.4872
resampledRad	**0.7767**	**0.5055**	0.8198	0.8421	0.7179

(b)					
Training data	rad2 Hold-out test-set performance				
	F2	CohensKappa	AUC	Sensitivity	Specificity
stableRad1	0.6757	0.2275	0.7605	0.7895	0.4872
Rad1	0.7353	0.4636	0.7794	0.7895	0.7179
Rad2	0.6250	0.4208	0.8151	0.6316	**0.7949**
avgRad	0.7075	0.3525	0.7901	0.7895	0.6154
unionRad	0.7522	0.3073	0.7659	**0.8947**	0.4872
intersectionRad	0.6522	0.1371	0.6356	0.7895	0.3846
resampledRad	**0.7843**	**0.5351**	**0.8381**	0.8421	0.7436

(c)					
Training data	resampledRad Hold-out test-set performance				
	F2	CohensKappa	AUC	Sensitivity	Specificity
stableRad1	0.6696	0.2042	0.7537	0.7895	0.4615
Rad1	0.7353	0.4636	0.7841	0.7895	0.7179
Rad2	0.6633	0.4358	**0.8192**	0.6842	**0.7692**
avgRad	0.6944	0.3008	0.7746	0.7895	0.5641
unionRad	0.7522	0.3073	0.7827	**0.8947**	0.4872
intersectionRad	0.6522	0.1371	0.6430	0.7895	0.3846
resampledRad	**0.7767**	**0.5055**	**0.8192**	0.8421	0.7179

One from each radiologist (subtables a and b) and one with resampled radiologists (subtable c). The highest value per column is highlighted in bold.

#### Statistical comparison of classifiers

The *p*-value results for the statistical comparison of the classifiers with FDR correction can be found in Table 2. As expected, there is a statistically significant difference between the higher performing classifiers, rad1, rad2, and resampledRad (approaches 2, 3, and 7, respectively), and the lowest performing classifiers stableRad1 and intersectionRad (approaches 1 and 6, respectively). Furthermore, the differences between training classifiers with rad1, rad2, or resampledRad data proved statistically non-significant. Therefore, the resampledRad dataset was selected for further analysis.

**Table 2:** *p*-value results with FDR correction for the statistical comparison of classifier performance on the resampledRad hold-out test set.

	stableRad1	rad1	rad2	avgRad	unionRad	intersectionRad
rad1	0.0631					
rad2	**0.0466**	0.7784				
avgRad	0.6003	0.1511	0.1799			
unionRad	0.7784	0.1624	0.1802	1.0000		
intersectionRad	0.7784	**0.0097**	**0.0223**	0.1624	0.2958	
resampledRad	**0.0321**	1.0000	1.0000	0.0969	0.0969	**0.0097**

p-values< 0.05 are highlighted in bold. The column and the row corresponding to resampledRad and stableRad1, respectively, have been removed due to not containing information.

**Figure 4:**
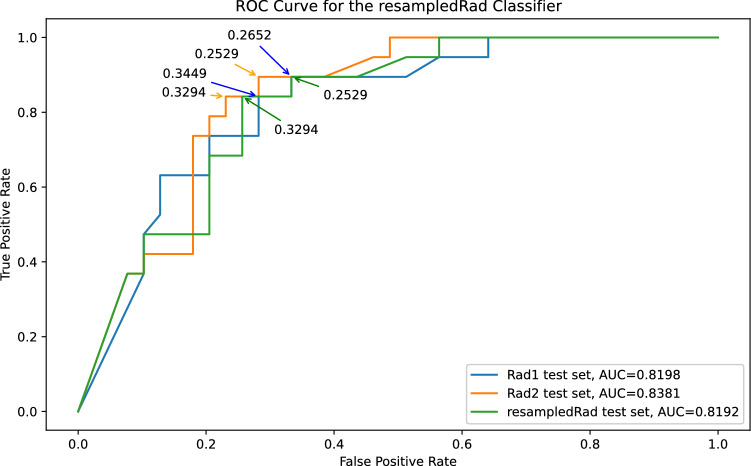
Receiver Operator Characteristics Curve for the resampledRad classifier when applied to the rad1, rad2 and resampledRad hold-out test sets, respectively in blue, orange and green. Some of the probability decision thresholds are included as annotations.

Figure 4 shows ROC curve for the resampledRad classifier. The decision threshold chosen for this classifier was 0.32, which ensured a minimum 0.9 sensitivity on the train set. Analysing Fig. 4, it is possible to conclude that the decision threshold would need to be lowered to at least 0.25 to reach a sensitivity of 0.9 across the test sets, providing evidence of overfitting.

#### Predictive variables selected

The resampledRad classifier utilizes 40 radiomic features, the majority of which was calculated from the ADC map (24/40 features). The T2W and DWI images contributed with 7 and 9 features, respectively. As far as image filters are concerned, gradient was the most substantial contributor with 12 features, this was followed by the original image with 6 features, and the remaining filters contributed between 1 and 4 features, with the exception of the square filter, which was the only one not represented. Regarding types of radiomic features, the most represented group is texture, with 28 features included. First-order and shape contributed with 9 and 3 features, respectively.

#### SHAP importance analysis

**Figure 5:**
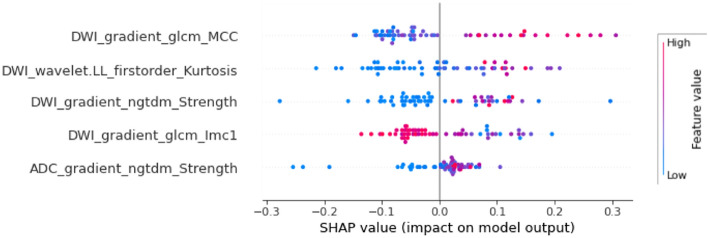
Summary beeswarm plot showing the five features with the highest impact on resampledRad model output according to a SHAP analysis explaining the model’s predictions for the resampledRad hold-out test set.

Regarding each feature’s contribution to model output, a shap analysis was performed, attempting to explain the predictions of each hold-out test set. The five features with the highest impact were consistent on the three hold-out test sets. Thus, as an example, the shap analysis for the predictions of the resampledRad classifier on the resampledRad test set is displayed in Fig. 5. The feature DWI_gradient_glcm_lmc1 is inversely associated with a clinically significant output, while the remaining four features are directly associated with it.

### Research question II

The deep and hybrid classifiers with the highest performance were selected. The deep features dataset that performed the highest was extracted from a Dense-2 Attention Unet and calculated through the max pooling approach, while the selected hybrid dataset was composed of resampledRad radiomic features and deep features extracted from a Dense-2 Unet and calculated through max pooling.

Comparing the classifiers trained with radiomic or deep features (Table 3), we can see that the classifier trained with deep features shows a higher cross-validation performance, however, the larger difference between CV and hold-out test performance indicates a larger degree of overfitting. These results are concordant with what is currently found in the literature^18^. Looking at the classifier trained with the hybrid dataset, we can see that it achieves a similar cross-validation performance to the other models. However, even though it is less overfitted, the classifier with the lowest degree of overfitting is still the radiomics one.

**Table 3:** 3-fold cross-validation, (a), and hold-out test set performance, (b), of classifiers trained with radiomic, deep and hybrid datasets.

(a)					
Training data	3-fold cross-validation performance (95% CI)				
	F2	CohensKappa	AUC	Sensitivity	Specificity
Rad	0.8830 (0.8129, 0.9570)	0.7350 (0.5300, 0.8562)	0.8679 (0.7654, 0.9281)	0.8920 (0.8164, 0.9762)	0.8439 (0.6697, 0.9163)
Deep	0.9577 (0.8267, 0.9675)	0.8609 (0.6684, 0.9235)	0.9311 (0.8338, 0.9615)	0.9753 (0.8135, 0.9762)	0.8870 (0.7701, 0.9639)
Hybrid	0.9392 (0.8236, 0.9892)	0.9164 (0.7175, 0.9444)	0.9583 (0.8585, 0.9722)	0.9306 (0.8056, 1)	0.9861 (0.8282, 0.9868)

(b)					
Training data	Hold-out test-set performance				
	F2	CohensKappa	AUC	Sensitivity	Specificity
Rad	0.7767	0.5055	0.8192	0.8421	0.7179
Deep	0.3333	0.2648	0.8131	0.2941	0.9355
Hybrid	0.5063	0.4062	0.8710	0.4706	0.9032

## Discussion

In the current study, radiomic and deep features where extracted from the whole prostate gland rather than the lesion. We attempted to answer the well-known issue of inter-reader variability introduced into the radiomics pipeline in the segmentation stage (RQ I). Finally, our study demonstrates the promising combination of radiomic and deep features for classifying prostate cancer disease aggressiveness (RQ II).

Publications extracting radiomic features from the whole prostate gland are rare, but there is evidence to support this method in contrast to single-lesion radiomics^42,43^. Previous work^43^ has reported that whole-gland radiomic features have higher stability to segmentation, since gland borders are easier to be defined comparing to a lesion’s, and that these features produced classifiers with higher performance and less overfitting. This might be justified by the existence of regions in the prostate, outside of lesions, that have relevant information for the classification and would not be easily visually recognized and subsequently segmented by the radiologist.

Radiomic features’ stability is essential for developing robust models to use in clinical scenarios. However, it is uncommon to find in the literature studies that assess this issue. From the studies that address it, most use the intraclass correlation coefficient to evaluate the agreement between readers and exclude features where a significant disagreement is found, training classifiers using purely ’stable’ features^44,45^. The issue with this approach is that a disagreement between the segmentation borders does not necessarily mean the resulting features would not be good predictors, it simply means the readers do not agree on what its value should be. Hence, new approaches to combining information from two or more radiologists are of high importance. In this study, we addressed this with research question I.

Regarding this first research question, our results revealed that approach 1, corresponding to the evaluation of feature stability through inter-rater absolute agreement and subsequent removal of unstable features, a technique currently recommended by radiomics guidelines and evaluated in the radiomics quality score^46^, proved to produce the classifiers with the least ability to generalize to hold-out data. On the other hand, approach 7, corresponding to training classifiers with a radiomics dataset where segmentation masks were randomly chosen from the two available radiologists, proved to be the highest performing across all hold-out test sets. Supporting the hypothesis that the more heterogeneous the training data the more generalizable the classifier may be on unseen data. Additionally, the performance of such classifier was very similar on the different hold-out test sets, indicating its robustness to radiologists with different years of experience. This was further confirmed by the performance on the resampledRad test set, which simulates a real-world clinical environment, where a deployed model would be used by several physicians. Thus, these results are extremely relevant for the clinical translation of AI models. As of right now, but still staying vigilant to further validation studies, the results suggest that gathering segmentations from different radiologists will produce classifiers that are more robust to slight differences in segmentation margins.

Regarding research question II, the few publications comparing deep learning and radiomics-trained classical machine learning on the same classification problem^18,19^ reported higher performance on the train set when using deep learning, but lower performance on the test set when compared to classical machine learning algorithms trained with radiomic features. In this work, deep learning’s natural tendency for overfitting was confirmed, both on the deep and hybrid classifiers. Even though the hybrid classifier showed a lot less overfitting than the deep model, it was not enough to outperform the radiomics classifier. Despite this, we feel this hybrid approach is worth exploring further with larger datasets and externally validated.

This study has several limitations. First, this was a retrospective study, so a multi-center prospective analysis should be carried out to validate these results and investigate the impact these predictive models have on patient outcome. Second, only T2W, DWI, and ADC sequences were used. Other sequences, such as dynamic contrast-enhanced MRI, could be worth exploring, however, since there are not consistently part of the MRI examination protocols they were not included in our models. Third, although the overall class imbalance was addressed through SMOTE upsampling of the minority class, we did not address the imbalanced nature of the anatomical location of lesions, with the large majority of lesions belonging to the peripheral zone. It would be interesting to investigate the model’s performance on the different anatomical zones independently. Fourth, using a publicly available dataset increased transparency but limited our access to clinical data, such as PSA levels, patient age, or PI-RADS score, which are fundamental components of a clinician’s assessment, but could not be included in our model. Fifth, proper assessment of real-world clinical performance is only possible through external validation. This important validation step will be addressed in future work. Finally, inherent to the Gleason system is the subjectivity of cancer grading, so we must keep in mind that the gold standard used in this study is subject to human error and inter or intra-observer variability. In addition, the definition of clinical significance might be based on more than the Gleason score alone, and variables such as tumour volume or tumour category might be relevant.

## Conclusion

In conclusion, the results presented in this study are extremely relevant to the clinical translation of AI models. Heterogeneous radiomics datasets where segmentation masks come from more than one radiologist produced classifiers with the highest generalization power. Additionally, the combination of radiomic and deep features in the classification of prostate cancer disease aggressiveness is studied. Here, we have shown promising results with the hybrid approach, which is worth exploring further with larger datasets.

## Supplementary Information

Supplementary Information.

## Data Availability

The datasets analysed during the current study are available in the Cancer Imaging Archive repository, https://wiki.cancerimagingarchive.net/pages/viewpage.action?pageId=23691656.
