# Supplementary material for: Value of handcrafted and deep radiomic features towards training robust machine learning classifiers for prediction of prostate cancer disease aggressiveness
Source: Sci Rep. 2023 Apr 17;13:6206. doi: 10.1038/s41598-023-33339-0 (PMC10110526; doi:10.1038/s41598-023-33339-0)
Supplement: Supplementary file 1 — Supplementary Information. [file 41598_2023_33339_MOESM1_ESM.docx]

Supplementary Material

# SM 1 – Excluded patients

| Prostatex0015 | Prostatex0074 | Prostatex0173 |
| --- | --- | --- |
| Prostatex0025 | Prostatex0101 | Prostatex0190 |
| Prostatex0031 | Prostatex0102 | Prostatex0199 |
| Prostatex0032 | Prostatex0107 | Prostatex0200 |
| Prostatex0038 | Prostatex0109 | Prostatex0201 |
| Prostatex0052 | Prostatex0116 | Prostatex0202 |
| Prostatex0054 | Prostatex0148 | Prostatex0203 |
| Prostatex0058 | Prostatex0152 |  |

# SM 2 – Model Characteristics

| **Classifier** | **Number of features** | **Training Hyperparameters selected during tuning** | | | | | | | | **Decision threshold** |
| --- | --- | --- | --- | --- | --- | --- | --- | --- | --- | --- |
|  |  | **LR penalty** | **LR C** | **RF bootstrap** | **RF min_samples_split** | **RF max_depth** | **RF min_samples_leaf** | **RF max_features** | **RF n_estimators** |  |
| stableRad1 | 40 | L2 | 1000 |  |  |  |  |  |  | 0.3738544 |
| rad1 | 30 |  |  | FALSE | 5 | 10 | 1 | auto | 100 | 0.4542857 |
| rad2 | 30 |  |  | FALSE | 2 | 10 | 2 | auto | 200 | 0.4446364 |
| avgRad | 20 |  |  | FALSE | 2 | 10 | 1 | auto | 200 | 0.2552381 |
| unionRad | 25 | l2 | 0.01 |  |  |  |  |  |  | 0.4280719 |
| intersectionRad | 10 |  |  | FALSE | 2 | 10 | 4 | auto | 200 | 0.3601615 |
| resampledRad | 40 |  |  | FALSE | 2 | 10 | 1 | auto | 200 | 0.3230612 |
| Deep | 35 |  |  | TRUE | 2 | 10 | 1 | auto | 200 | 0 |
| Hybrid | 30 |  |  | TRUE | 5 | 10 | 1 | auto | 200 | 0.4905615 |
